# Data storage mechanism of industrial IoT based on LRC sharding blockchain

**DOI:** 10.1038/s41598-023-29917-x

**Published:** 2023-02-16

**Authors:** Yongjun Ren, Xinyu Liu, Pradip Kumar Sharma, Osama Alfarraj, Amr Tolba, Shenqing Wang, Jin Wang

**Affiliations:** 1 Engineering Research Center of Digital Forensics, Ministry of Education, School of Computer Science, Nanjing University of Information Science and Technology, Nanjing, 210044 China; 2 Department of Computing Science, University of Aberdeen, Aberdeen, AB24 3FX UK; 3 Computer Science Department, Community College, King Saud University, Riyadh, 11437 Saudi Arabia; 4 College of Computer Science and Technology, Nanjing University of Aeronautics and Astronautics, Nanjing, 211106 China; 5 School of Computer and Communication Engineering, Changsha University of Science and Technology, Changsha, 410114 China

**Keywords:** Computer science, Information technology

## Abstract

With the rapid development of Industry 4.0, the data security of Industrial Internet of Things in the Industry 4.0 environment has received widespread attention. Blockchain has the characteristics of decentralization and tamper-proof. Therefore, it has a natural advantage in solving the data security problem of Industrial Internet of Things. However, current blockchain technologies face challenges in providing consistency, scalability and data security at the same time in Industrial Internet of Things. To address the scalability problem and data security problem of Industrial Internet of Things, this paper constructs a highly scalable data storage mechanism for Industrial Internet of Things based on coded sharding blockchain. The mechanism uses coded sharding technology for data processing to improve the fault tolerance and storage load of the blockchain to solve the scalability problem. Then a cryptographic accumulator-based data storage scheme is designed which connects the cryptographic accumulator with the sharding nodes to save storage overhead and solve the security problem of data storage and verification. Finally, the scheme is proved to be security and the performance of the scheme is evaluated.

## Introduction

Industrial Internet of Things (IIoT) is the continuous integration of various types of acquisition and control sensors or controllers with sensing and monitoring capabilities, as well as mobile communication, intelligent analysis and other technologies into various aspects of the industrial production process, thereby significantly improving manufacturing efficiency, improving product quality, reducing product costs and resource consumption, and ultimately achieving a new stage of upgrading traditional industry to intelligence^1,2^. IIoT as an emerging product, the architecture is more complex, there is no unified standard, all aspects of security problems are more prominent^3,4^.

Among them, the data security of the IIoT is facing great challenges. Blockchain has the characteristics of decentralization and tamper-proof modification, and has natural advantages in solving the data security problems of the IIoT. The blockchain is layered through a peer-to-peer (P2P) network so that the whole network can perform complete information transmission and verify its accuracy^5–9^. In addition, the blockchain uses automatic filtering mode to establish credit information. This reliable information can effectively improve the security of IIoT transactions. More importantly, blockchain nodes can participate or leave independently without any interference to the whole blockchain^10–13^. Therefore, the blockchain solution can reasonably integrate networking data resources and improve the security of IIoT users.

However, because the current blockchain can’t meet the dilemma of data security and scalability of IIoT, and the blockchain^14–17^ itself still has security problems of data storage and verification, this paper integrates the coded sharding blockchain into IIoT. Therefore, the specific contributions of this paper are as follows:

1. This paper studies the requirements of IIoT and analyzes the challenge of ensuring the scalability and data security of IIoT system while providing consistency with blockchain.
2. According to the requirements of system scalability and data security of IIoT, an IIoT storage architecture based on local repairable code (LRC) sharding blockchain is constructed by combining coded sharding blockchain with IIoT.
3. This paper shows the scheme of connecting the bilinear accumulator with the shard node, using the accumulator as the storage structure for data storage. The functions of membership witness and membership verification of the accumulator can solve the data storage and verification security problems existing in the blockchain itself.

## Results

### System structure

**Figure 1:**
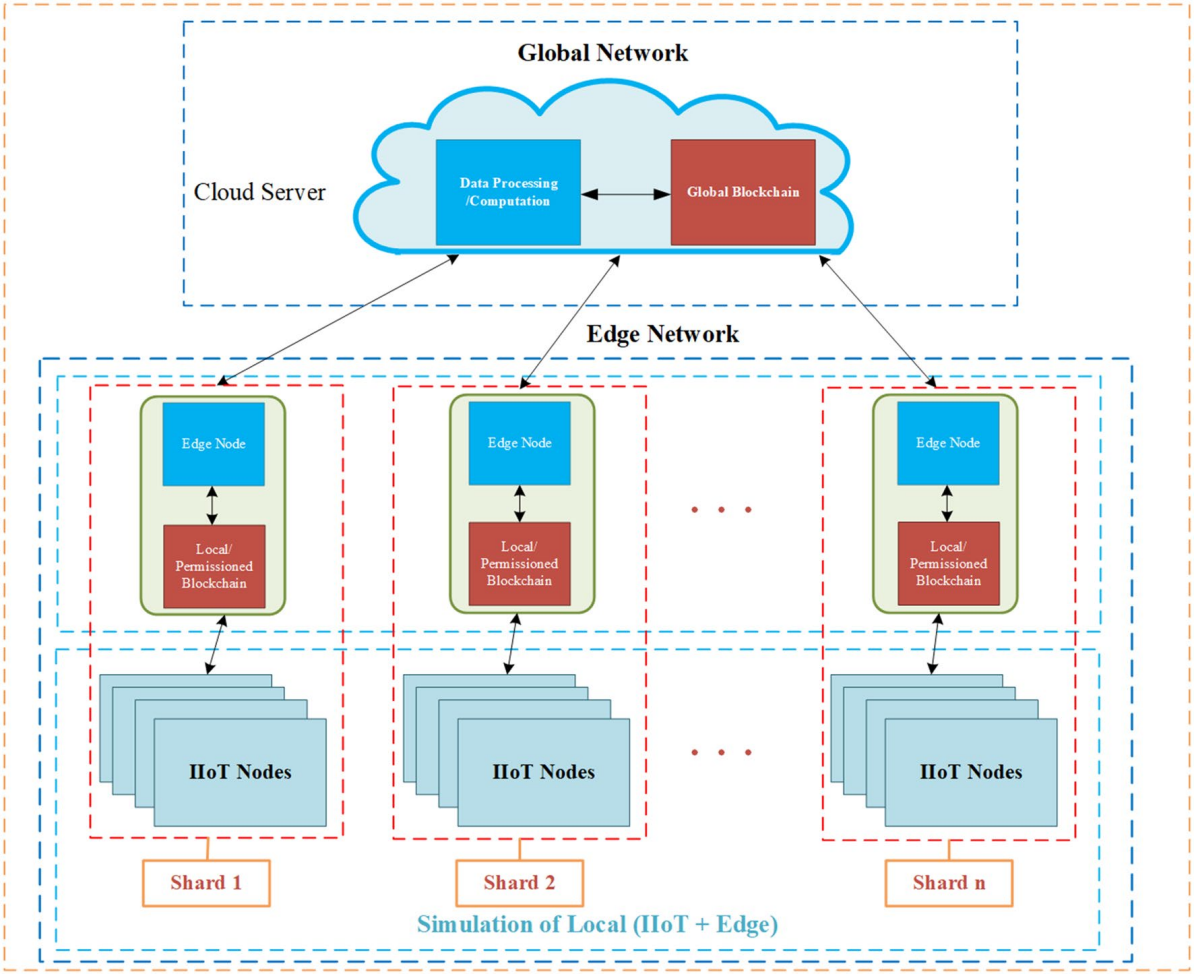
Overall system structure diagram.

The IIoT network system shown in Fig. 1, which uses a sharding blockchain and consists of two main elements:

#### Global network

Global/centralized networks can provide the highest resource capacity. It follows the traditional centralized cloud computing method, consists of cloud servers that host software, is responsible for planning, monitoring and managing resources, and handles the logical execution of architectural functional components, and it provides a globally available service platform for applications that require high storage and computing power^18–20^. As the core architecture of IIoT, it is connected with the blockchain to form the overall system structure of the combination of IIoT and blockchain.

#### Edge network

The blockchain is connected through a global network as an edge network^21–23^. Each blockchain contains n shards, and each shard contains an edge node and an IIoT node that can be stored in the licensed blockchain, and the two nodes are connected to form a shard node. Finally, the edge network is connected with the global network to form the overall structure shown in Fig. 1. In order to further improve the system structure formed by IIoT and the sharding blockchain, we add the LRC to solve the IIoT data reliability problems, and add bilinear accumulator to solve the data storage and verification security problems of the blockchain itself. The structure realizes data storage security through bilinear accumulator, and uses coded sharding technology to ensure data reliability and blockchain scalability in the process of operation.

In the system structure, the coded sharding technology ensures the data reliability of IIoT and increases the fault tolerance of the blockchain system. At the same time, the anti-attack ability of the system can be increased by encoding the data.

### LRC sharding blockchain structure

In order to better ensure the data security of IIoT, we propose to encode the data blocks in each shard node of the blockchain. This kind of sharding coded is used to ensure the reliability of the data.

**Figure 2:**
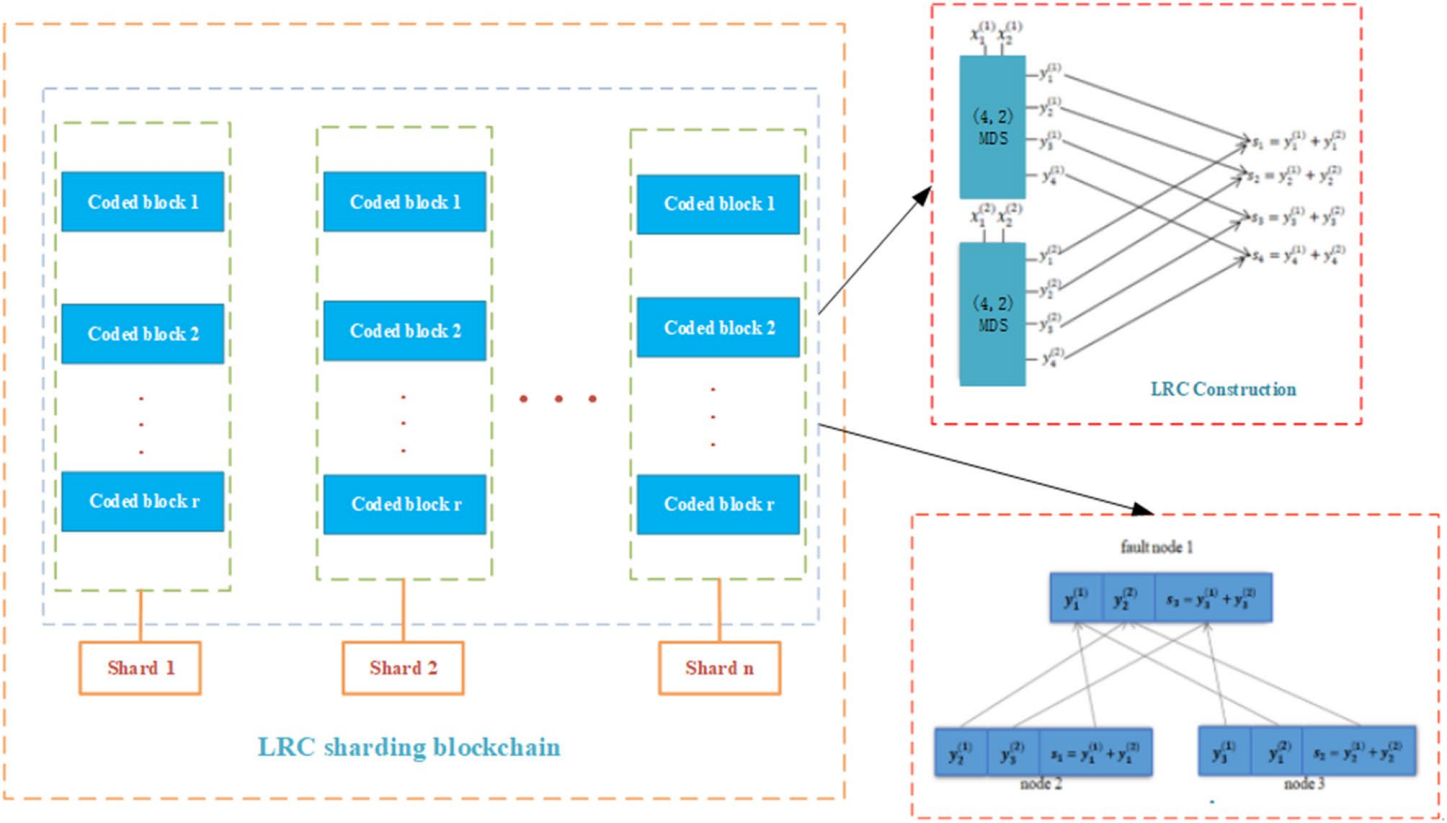
LRC sharding blockchain structure.

The LRC can not only ensure the reliability of the data in the blockchain, but also repair the wrong node data, which has lower bandwidth overhead and higher repair efficiency. Therefore, we choose the LRC to encode the node data blocks after the blockchain is sharded^24^. LRC sharding blockchain (LRC-SB) structure as shown in Fig. 2.

## Discussion

No blockchain system can achieve consistency, security, and performance scalability at the same time. For the current blockchain system, as more nodes join the network, the efficiency of the system remains constant at most. One of the main ways to achieve blockchain scalability is to use the concept of shard^10,25,26^ : It divides the network into multiple regions, which process the corresponding transactions in parallel. In the underlying public blockchain system, the transactions on the blockchain will be divided into different pieces, which are composed of different nodes on the network. Therefore, only a small part of the input transactions needs to be processed, and a lot of verification work can be done by processing in parallel with other nodes on the network. Dividing the network into fragments allows more transactions to be processed and verified at the same time, so in the blockchain, a single blockchain can be split into multiple sub-chains running in parallel, and different sub-chains deal with different parts of the blockchain, thus reducing the load on each individual node. However, the existing distribution proposal may damage the nodes in a given shard and cause permanent damage to the corresponding part, so as to achieve efficient scaling. In^27^, the “polynomially coded sharding” scheme is proposed to realize the scalability and trust of the blockchain system. Although this method can also solve the security problems caused by shard. However, the security problem of data storage and verification in the blockchain itself has not been solved. Compared with the existing scheme^28^, the bilinear mapping accumulator is faster than the RSA accumulator and performs better than expected in most cases where accumulators are needed. Therefore, this paper uses bilinear accumulator to solve the security problem of data storage and verification of blockchain.

A large amount of data needs to be stored and processed in IIoT. In order to improve the security and scalability of the current IIoT, this paper proposes to use local repairable code (LRC) sharding technology, combined with bilinear accumulator to solve this dilemma. Compared with the polynomially coded sharding technology, LRC sharding technology has better scalability and efficiency, because the data repair can be completed in the local network, reducing the communication cost of data repair. Therefore, we use local repair code (LRC) sharding technology to replace the “polynomially coding sharding” used in^27^ to ensure the scalability of the system, but the security problems of data storage and verification in the blockchain itself have not been solved, so we use bilinear accumulators to improve data security. Scalability essentially refers to the throughput of the system, in which the best way to improve throughput is shard technology. The main idea is to divide the blockchain into several independent sub-chains, and then process the data on each sub-chain separately. However, it is easy to have data error or node data loss, so we combine the LRC and shard technology, which have great advantages in repairing the error node data, to improve the throughput of the system on the basis of ensuring data integrity. Although the use of LRC^29–31^ can ensure the integrity and correctness of data to a certain extent, it can’t completely resist enemy attacks and ensure the security problems encountered in the process of data transmission. Therefore, we use the bilinear accumulator, which is widely used and efficient, in the sharding blockchain, connect the accumulator with the shard node, and use the accumulator as the storage structure for data storage. The functions of membership witness and membership verification of the accumulator are used to ensure the data security of the blockchain.

This paper proposes a data storage mechanism of IIoT based on LRC sharding blockchain, which not only ensures the reliability of IIoT data, but also increases the fault tolerance and anti-attack ability of the blockchain system. However, the operation process of adding a cryptographic accumulator to the blockchain is relatively complex to store data directly in the blockchain. In this process, we need to further verify the data, it can better ensure the security of blockchain data storage and verification, so how to simplify the operation flow of the system structure needs to be further discussed.

## Methods

### LRC construction

Let the node *x* with the size of $M=r k$ data be divided into *r* parts, $x=\left[ x^{(1)}, \ldots , x^{(r)}\right]$, where each $x^{(i)}, i \in [r]$, and the size is *k*. We use the extremal (*n*, *k*, *d*)-MDS (maximum distance separable code) code $y^{(1)}=x^{(1)} G, \ldots , y^{(r)}=x^{(r)} G$, where *G* is the $n \times k$ MDS generating matrix, and each part of the *r* part is independently encoded into a coding vector $y^{(i)}$ of length *n*, where $(r+1) \mid n$.

As a MDS precoding^32^, we use (*n*, *k*)-RS (Reed–solomon) code, which requires that every element of *k* is over a finite field $F_{2^p}$, such that $2^p \ge n$ for any *p*. This will mean that all the sub data stored in our coding is on finite field with size $2^p \ge n$. Afterwards, a single parity $\textrm{XOR}$ vector $S=\bigoplus _{i=1}^r y^{(i)}$ is generated from the coding vectors.

The precoding process produces a total *r*.*n* coding blocks, $y^{(i)}$ vectors and *n* $\textrm{XOR}$ parity blocks in *s* vectors. Means of the total community $(r+1). n$ blocks can be placed in *n* nodes, so we decide that each node stores $r+1$ block. Therefore, each node needs to have the following storage capacity$\alpha =\frac{M}{k}+\frac{1}{r} \frac{M}{k}=r+1($ coded blocks ).

According to the construction of the LRC, we can conclude that the specific algorithms for LRC to encode and decode the data in the shard are shown in Algorithms 1 and 2.
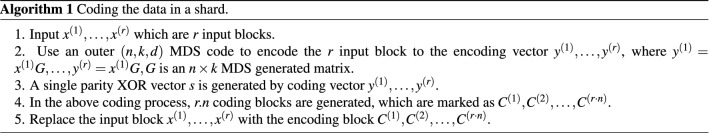

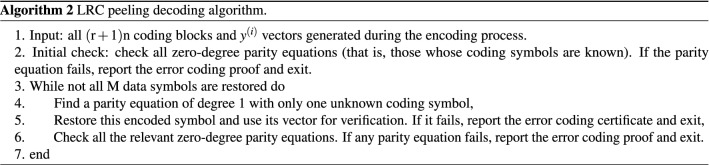


### LRC fault shard node repair

To repair each missing node, it is necessary to contact *r* nodes, that is, the existence of locality is the code of *r*. In the case of general non-lost cases, the repair of nodes in the first repair group in $r+1$ node needs to be considered. It is enough, because the node follows the put attribute of the same in different repair group.

The main observation is that each node stores a block with a different index of $r+1$ in the repair group: the $r+1$ blocks of a specific index are stored in $r+1$ different nodes within a single repair group. Such as, when the first node is faulty, you need to download $s_1$ in the second node, download $y_1^{(r+1)}$ to regenerate the symbol $y_1^{(1)}$ of first row in the third node, and the rest may be deduced by analogy. Once all symbols are downloaded, $y_1^{(1)}$ is just a simple XOR from these symbols. By the same manner, when reconstructing the node in each repair group, first download the remaining identical index block, and then XOR them together, and finally regenerate the required lost blocks. Because of the reconstruction of each block needs to contact *r* other blocks, and can only be repaired in a single repair group with *r* remaining nodes, therefore the encoding has locality *r*.

### Data storage and verification of LRC-SB based on bilinear accumulator

The shard technology improves the throughput and scalability of the blockchain system, and LRC improves the data reliability of the blockchain. In the coded sharding blockchain, LRC is used to encode the data in the IIoT node to form redundant data, and the data is stored in the whole node of the corresponding shard of the edge network. However, the traditional blockchain must verify the whole node of the stored data and store all the data and membership witnesses in the node together, so the cost of running the full node and independent verification blockchain is very high, and there is no guarantee that the validated data is available and there is no hidden malicious data. Therefore, the verification efficiency of the original blockchain is very low, the node storage pressure is also very high, and cannot guarantee the availability of data^33,34^. Therefore, on the basis of LRC-SB, we propose to use pairing-based accumulator to solve these problems.

#### Construction of bilinear accumulator

The cryptographic accumulator scheme allows the finite set $X=\left\{ x_1, \ldots , x_n\right\}$ to be accumulated into the accumulative value ${\text {acc}}_X$, the so-called accumulator. For each element $x_i \in X$, the so-called witness $w i t_{x_i}$ can be effectively calculated to prove membership of $x_i$ in $a c c_X$. Based on the $n-\textrm{SDH}$ assumption of the bilinear pair accumulator^35–37^, works as follows:

1. ${\text {Gen}}\left( 1^k, t\right)$ : Input the security parameter *k* and select the three groups $G_1, G_2$ and $G_T$ with prime order *p* to generate bilinear pairs $e: G_1 \times G_2 \rightarrow G_T$. Among them, the generator of $G_1, G_2$ is $g_1, g_2$, and $s \leftarrow Z_p^*$ is selected at the same time. Finally, the algorithm returns a set of keys $\left( s k_{a c c}, p k_{a c c}\right) \leftarrow \left( s,\left( g_1, g_1^s, \ldots , g_1^{s^t}\right) \right)$.
2. ${\text {Eval}}_r\left( \left( s k_{a c c}, p k_{a c c}\right) , X\right)$: Input the key pair $\left( s k_{a c c}, p k_{a c c}\right)$ and the set $X=\left\{ x_1, \ldots , x_n\right\}$. If $s k_{a c c}$ exists, the accumulative value of set *X* is calculated as follows: $a c c_X=g_1 \prod _{i=1}^n\left( x_i+s\right)$. If $s k_{a c c}$ is unknown, the accumulative value of set *X* cannot be calculated directly. So, if $\prod _{x \in X}(x+s)$ is calculated and expressed as $\sum _{i=0}^n a_i \cdot s^i$, the accumulative value of set *X* can be calculated as follows: ${\text {acc}}_X=\prod _{i=0}^n\left( g_1 s^i\right) ^{a_i}$. Finally, the algorithm returns the accumulative value acc $c_X$ and the auxiliary value $a u x=(X)$.
3. WitCreate $\left( \left( s k_{a c c}, p k_{a c c}\right) , a c c_X, a u x, x_i\right)$: Input the key pair $\left( s k_{a c c}, p k_{a c c}\right)$, accumulative value $a c c_X$, auxiliary value $a u x=(X)$ and element $x_i$. The algorithm first verifies whether element $x_i$ belongs to set *X*. If not, return $\perp$. If $s k_{a c c}$ exists, the witness of element $x_i$ can be calculated as follows: wit $_{x_i}=a c c_X^{\left( x_i+s\right) ^{-1}}$. If the $s k_{a c c}$ is unknown, the $w i t_{x_i}$ cannot be calculated directly. But we can calculate $\prod _{x \in X \backslash x_i}(x+s)$ and represent it as $\sum _{i=0}^{n-1} a_i \cdot s^i$, then the witness of element $x_i$ can be calculated as follows: wit $_{x_i}=\prod _{i=0}^{n-1}\left( g_1^{s^i}\right) ^{a_i}$.
4. ${\text {Verify}}\left( p k_{a c c}, {\text {acc}}_X\right.$, wit $\left. _{x_i}, x_i\right)$ : The algorithm determines whether element $x_i$ is a member of set *X* by verifying that $e\left( {\text {acc}}_X, g_2\right) =e\left( \right.$ wit $\left. _{x_i}, g_2 x_i g_2^s\right)$. If the above formula is true, the algorithm returns true, otherwise it returns false.

#### Storage and verification scheme description

**Figure 3:**
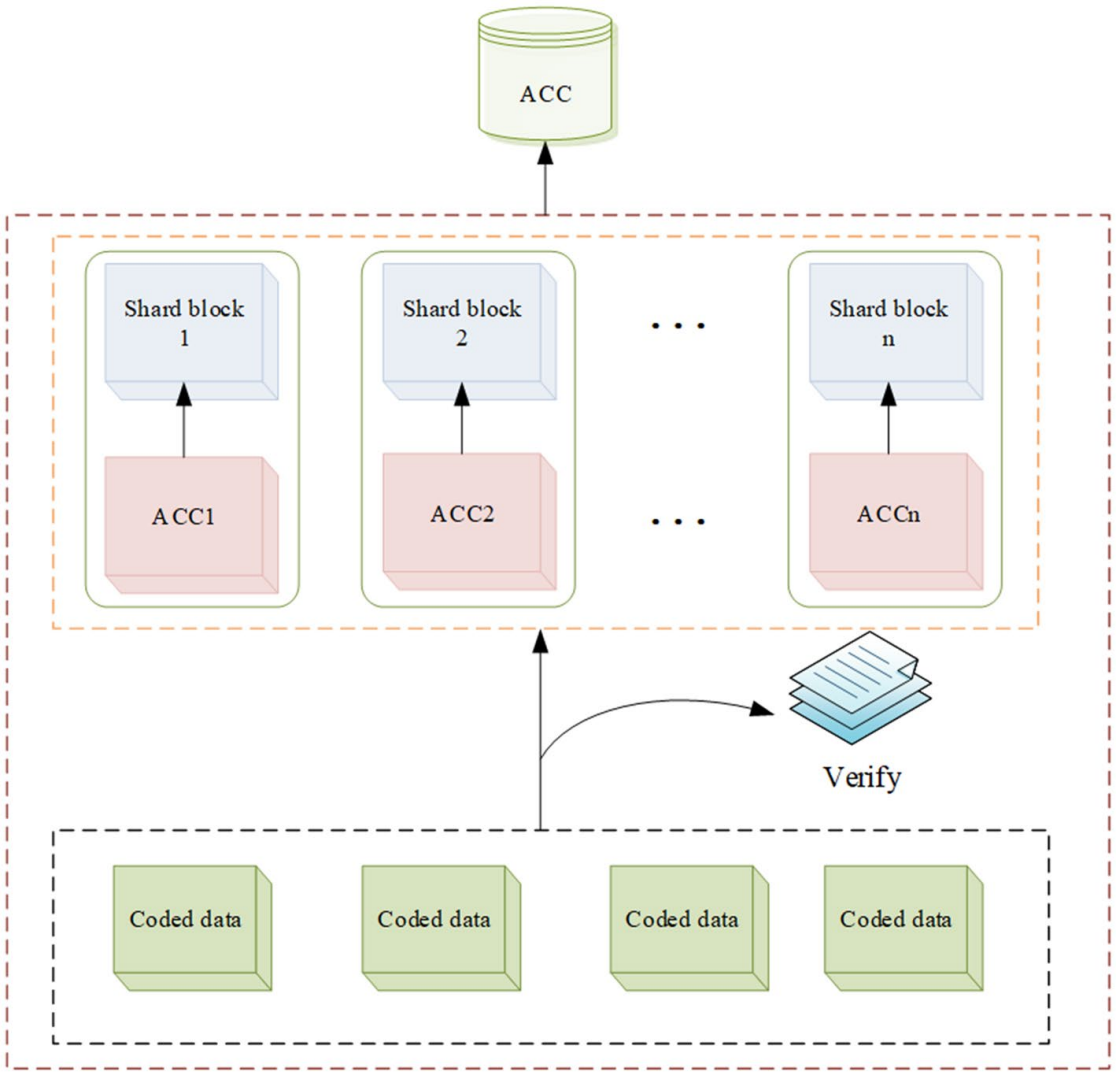
Coded sharding blockchain storage scheme based on bilinear accumulator.

As shown in Fig. 3, each shard constructs an accumulator, and the blocks in each shard are connected to the accumulator, and at the same time, each block contains a new accumulator to compress and store the encoded available data, and then validate the data to ensure that the data stored in the block is available.

All the shard full nodes are connected to an accumulator, and there is also an accumulator in the block of each shard node for data storage and data verification^38^, it only needs to store the data that is considered to have data availability after verification. Because the encoded data is already resistant to attack, it is difficult for malicious nodes to hide some data in the encoded data, so it is difficult for attackers to hide some data in the shard nodes, then use bilinear accumulators to provide membership witness (CodeMemWitCreate) for the encoded data, and use VerifyCodeMem algorithm to authenticate the encoded data to ensure the availability of these data.

The whole node after shard can use the BatchAdd algorithm to add the encoded data with data availability verified to the node, and for the unencoded data or the discovered malicious hidden data, if it has been added to the shard node, it can be deleted using the BatchDel algorithm. The NonCodeMemWitCreate algorithm is used to provide non-membership witness for the unencoded data or the discovered malicious hidden data, and the VerifyCodeNonMem algorithm is used for non-membership witness. All the algorithms mentioned above are shown in Algorithm 3 and the parameters required for the above algorithm are shown in Table 1.

**Table 1:** Algorithm parameters.

$\lambda$	Security parameters
$A_t$	A discrete time counter
$D=\left\{ d_{1, \ldots ,} d_n\right\}$	Accumulator value at time t
$D_0$	Current accumulated elements set
*m*	A subset of the set D
$w_x^t$	Information for updating certificates
$u_x^t$	Membership witness
Bezout (*x*, *y*)	The subprocess of outputting the Bezout coefficient $a, b \in Z$ for a pair of integers *x*, *y* (that is, satisfying the relation $ax+by=1$ ) which are primes to each other

The bilinear accumulator is added to the encoded blockchain, so that the shard node only needs to store the encoded node and verify the encoded node, so it can reduce the data storage burden of the traditional blockchain to the node and improve the verification efficiency of the node, and then combined with coding to reduce the malicious hidden data of the attacker while providing membership witness for the encoded data. Further solve the problem of data availability of blockchain.
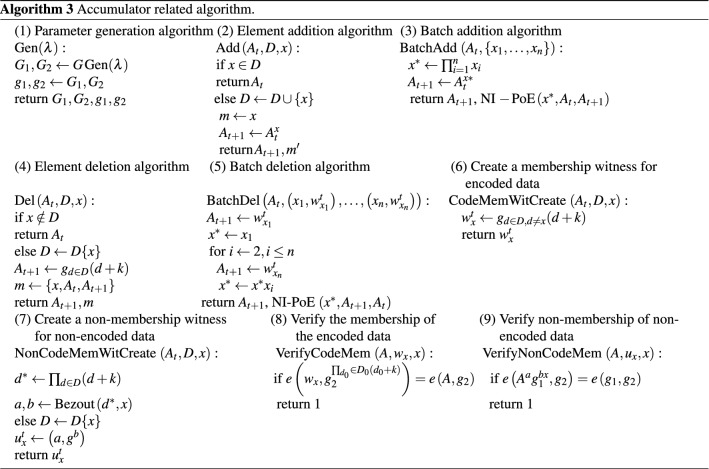


#### Adversary model

The data of IIoT is stored on several untrusted nodes of the blockchain system. We consider an isomorphic synchronous network, that is, all nodes have similar processing power, and the communication delay between any pair of nodes is bounded by a known constant. Some nodes may be corrupted and affected by Byzantine failures, that is, they may calculate and transmit arbitrarily incorrect results during block verification. Our goal is to design a secure verification scheme for the following strong adversary models:

1. Attackers can destroy a fixed proportion of network nodes, that is, the number of malicious nodes increases linearly.
2. If a traditional sharding solution is used, the attacker knows the node to shard allocation and can adaptively select the node to attack.

We note that under this opponent model, the random sharding rotation method is no longer secure, because after knowing which nodes are assigned to the shard, the adversary can concentrate on attacking a single shard. Therefore, we use the coding sharding technology in the system model, which can better ensure the reliability of the data after the sharding is attacked, even if the encoded data is attacked, it is difficult to obtain the correct data information. Then the membership witness function of the bilinear accumulator is used to ensure the data verification security of the system.

In the data storage process of blockchain, the system is vulnerable to data availability attacks, and coded sharing technology can better ensure data reliability. Any small hiding on the original block caused by the encoded data will be tantamount to making a large part of the coded block unavailable, which can be detected by the node by exponentially increasing the probability of random sampling of the coded block.

#### Security analysis

The data storage and verification scheme of the coded sharding blockchain in IIoT is exposed to various attacks. We will show how we propose how to defend these possible attacks.

##### Data delete attack

If the shard blockchain is an edge network that deletes and destroys the original data, the witness of the encoded data block cannot be calculated: $w_x^t \leftarrow g_1^{\prod _{d \in D, d \ne x}(d+k)}$.

When it receives the verifier verification. Since each encoded data block in the sharding blockchain is a coded accumulated value $A_t$, the IIoT global network cannot only use original data to generate a valid ownership certificate.

##### Replacement attack

If the IIoT edge network replaces damaged or deleted data with other valid encoded data blocks, and when the verifier uses a random block witnessed $w_x^{'\text {t }}$ to verify the encoded data block in the edge network of IIoT, the verifier is calculated witness $w_x^{'\text {t }}$ cannot match the extracted member witness $w_x^t$.

##### Data hiding attack

The data owner uses the local repair code to encode the data in the node to generate encoded data block $C^{(1)}, C^{(2)}, \ldots , C^{(r \cdot n)}$. Because the encoded data is resistant to attack, it is difficult for malicious nodes to hide in the encoded data, so the possibility of attackers hiding some data in shard nodes is almost negligible.

##### Replay attack

It makes no sense for cloud service providers to cache witnesses from past computing to meet new challenges from current validators. On the one hand, the cloud service provider cache witness needs to store both the target block and the corresponding witness, which will greatly increase the storage overhead of the cloud service provider. On the other hand, the verifier challenges the cloud storage provider with the cumulative membership of random data blocks when verifying data integrity, so the probability of the same challenge is basically negligible.

##### Data disclosure attack

In the setup phase, the data owner accumulates each encoded data block using a bilinear accumulator, and the data accumulated in the accumulated set has its own membership proof. As a result, others cannot know the data outsourced to IIoT cloud service providers.

##### Double spending attack

All data needs to be compressed and stored through a bilinear accumulator, and membership verification is required in the stored process. Even if a 51% attack is launched to copy the same data for operation, membership verification is required. However, the data already stored in the cryptography accumulator will not be added again, so the purpose of the double spending attack will not be achieved.

## Experimental and analysis

### Performance evaluation

We use Aliyun and Amazon S3. Compared with a single cloud storage service, the response time of a distributed cloud storage service composed of multiple cloud storage services is more stable and faster. This is because the cloud storage based on blockchain provides multiple copies of data and can take advantage of the network bandwidth of multiple cloud storage services to overcome the bandwidth shortage of a single cloud server.

In the case of replica repair based on blockchain, when local repair codes are used to repair faulty data, the repair bandwidth and disk I/O will be reduced. As the redundancy m increases, it helps to repair the decrease in bandwidth and disk I/O. In view of the fact that our system can significantly reduce the amount of storage required by nodes without significantly increasing CPU decryption, running the blockchain on IIoT devices is a very important step.

The central goal of the system is scalability. Let $\textrm{N}$ be the number of nodes and $\textrm{b}$ the block size. Consider the simplest extension solution, which is to spread data across the network without duplication. Storage overhead measures the ratio of the total storage cost to the actual storage information. Consider that everyone keeps a complete copy of the data and the storage overhead is $\textrm{O}(\textrm{N})$, which indicates that the storage cost increases linearly with the size of the network. LRC sharding blockchain realizes $\textrm{O}(1)$ storage in real time on the client side, and $\textrm{O}(\textrm{Logb})$ storage in case of client damage. When using renewable code, the worst-case scenario is that the opponent sends the wrong block prediction node that needs to download $\textrm{O}(\textrm{b})$ data to prevent fraud. LRC sharding blockchain achieves near-optimal overhead and only needs to download $\textrm{O}(\textrm{Logb})$ proof.

We measure the amount of data stored by each node after 1500 blocks (about 6 million transactions) are processed in the LRC sharding blockchain. Compared to previous work, we estimated the storage space required for each node in PolyShard and RC-blockchain based on the reported throughput and the number of shards of similar network size, as shown in Table 2.

**Table 2:** Storage required for each node after 5 million transactions have been processed.

Protocol	Network size	Storage
PolyShard	1200 nodes	500 MB
RC blockchain	1200 nodes	700 MB
LRC-SB	1200 nodes	238 MB

### Performance analysis

#### Bandwidth consumption during sharding node coding

**Figure 4:**
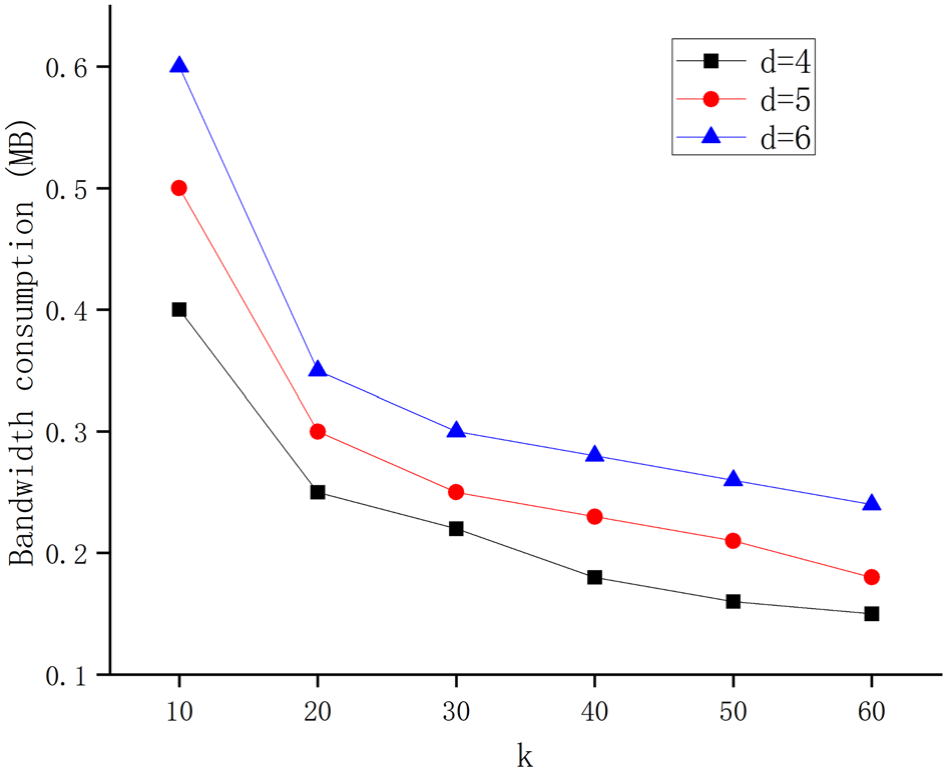
Bandwidth consumption when sharding nodes are encoded.

In each shard, we can calculate the bandwidth consumed by the node in the coding process. We evaluate the bandwidth consumption of each node in which each shard node stores *d* encoding fragments. The bandwidth consumption of the nodes in the shard changes when coding at *k*=10, 20, 30, 40, 50, 60 and *d* = 4, 5, 6, where *k* represents the total number of coding fragments. Figure 4 shows the shard node storing code fragment in the process of encoding bandwidth consumption. It can be seen that when *d* is fixed, *k* is, the greater, the less bandwidth is consumed to store encoding fragments in the process of node coding. When *k* is fixed, *d* is larger, the greater the bandwidth consumption of storing encoded fragments in the coding process is. Therefore, the bandwidth occupied by the node for data transmission in the shard is related to the amount of coded data allocated to the node. When the block size is relatively large, the bandwidth consumption and the amount of data stored can be measured, and a better scheme can be chosen. However, with the current mainstream blockchain block size, the bandwidth occupied by data transmission between nodes in the packet is very small.

#### Error node repair rate of LRC

**Figure 5:**
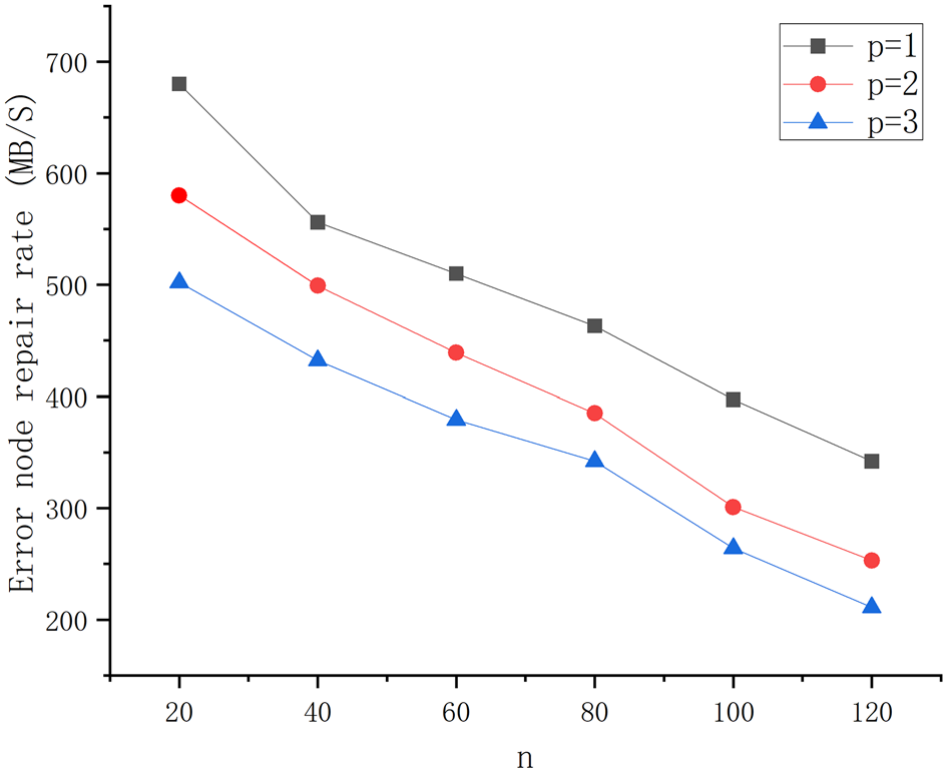
Error node repair rate.

In the experiment, we calculated the repair rate of the error nodes in each shard and evaluated the repair rate of the error nodes in the number of error nodes respectively. The total amount of encoded data of the nodes in the slice is the repair rate of the error nodes. Figure 5 shows the change in the repair rate of the error node. We can know that when *p* is fixed, the larger *n* is, the slower the repair rate of error nodes in each shard is. When *n* is fixed, the smaller *p* is, the faster the repair rate of error nodes is.

#### Performance analysis of LRC-SB

In order to better demonstrate the performance advantages of LRC-SB, we compare it with unencoded blockchains and renewable code-based blockchains^39,40^. Table 3 shows in detail the efficiency comparison of LRC-SB, RC blockchain and traditional blockchain in terms of throughput, storage efficiency and security, so as to show the advantages of LRC sharding.

**Table 3:** Comparison of performance and efficiency of three kinds of blockchains.

Metrics	Blockchain	RC blockchain	LRC-SB
Throughput	*O*(*n*)	*O*(*n*)	*O*(1)
Storage efficient	$O\left( n^2\right)$	$O(n \log n)$	*O*(*n*)
Security	*O*(*n*)	*O*(*logn*)	*O*(1)

**Figure 6:**
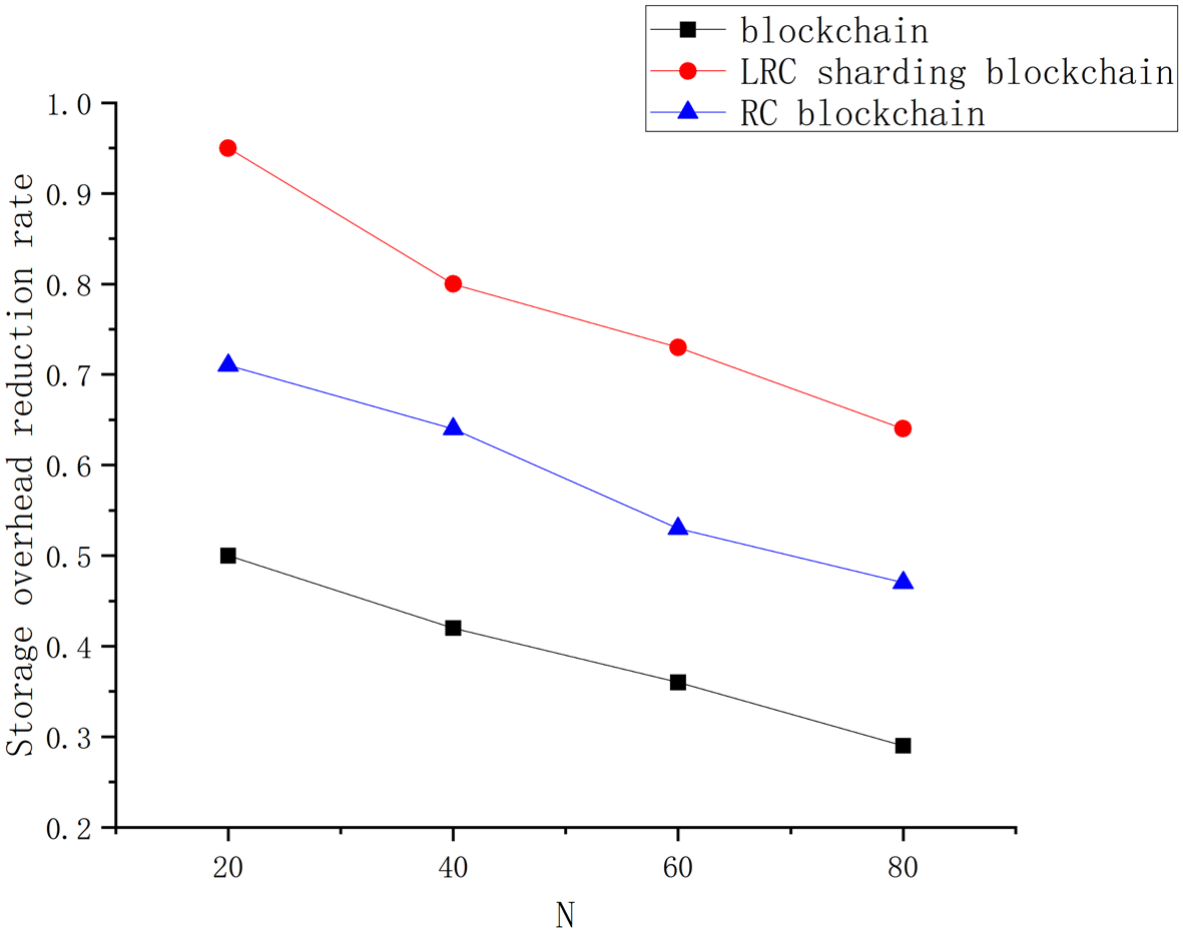
Reduction rate of storage overhead.

In order to reduce the storage pressure of the node, the LRC-SB stores the encoded data with the help of an accumulator in the shard node, so the storage efficiency of the blockchain is specially tested. By comparing the storage efficiency of the traditional blockchain and the RC blockchain, we find that the storage efficiency of the LRC-SB is greatly improved after adding the accumulator. By comparing the changes in the reduction rate of storage overhead among the three, the result is shown in Fig. 6.

### Analysis and comparison of system security

In order to better demonstrate the performance advantages of LRC-SB, we compare it with unencoded blockchains and renewable code-based blockchains, and analyze the differences among them in terms of throughput, latency, scalability, fault tolerance and security. The results are shown in Table 4.

**Table 4:** Comparison of performance and efficiency of three kinds of blockchains.

Metrics	Blockchain	RC blockchain	LRC-SB
Throughput	Around 1000 tps	Around 1000 tps	More than 1500 tps
Latency	Around 100 seconds	Around 100 seconds	Around 80 seconds
Scalability	Keep constant	Coding improves scalability	Coded sharding improves scalability
Fault tolerance	Unaffected	Affected by code repair rate	Affected by code repair rate
Security	Vulnerable	Anti-attack	Anti-attack

## Conclusion

The Industrial IoT provides great convenience for industrial development through data exchange and integrated control. However, the data security problem of the IIoT still exists. Blockchain has great potential in dealing with data security issues of the IIoT. However, blockchain has certain scalability problems and cannot meet the scalability requirements of IIoT. To solve the problem, this paper uses shard technology to solve the scalability of IIoT system. And the local repairable code technology is utilized to encode the data in the sharding blockchain to enhance the reliability of the data. Moreover, this paper connects the accumulator with the shard node and uses the accumulator as a data storage structure. The function of membership witness and membership verification of the accumulator can ensure the data security of the blockchain and solve the security problems of data storage and verification in the blockchain itself. Meanwhile, the data reliability and availability of the blockchain are also improved by using LRC sharding technology and accumulators.

In the blockchain system, data security and privacy are always a tradeoff^41,42^. In order to solve the problem of data security and privacy of blockchain at the same time, we intend to improve the cryptographic accumulator in the future research work, and use the cryptographic accumulator with zero knowledge to realize the tradeoff between data security and privacy of blockchain.

## Data Availability

The datasets generated and analyzed during the current study are not publicly available due to restricted data sources but are available from the corresponding author on reasonable request.
